# Stem cells labeled with superparamagnetic iron oxide nanoparticles in a preclinical model of cerebral ischemia: a systematic review with meta-analysis

**DOI:** 10.1186/s13287-015-0015-3

**Published:** 2015-03-13

**Authors:** Leopoldo P Nucci, Helio R Silva, Viviana Giampaoli, Javier B Mamani, Mariana P Nucci, Lionel F Gamarra

**Affiliations:** Hospital Israelita Albert Einstein, Av. Albert Einstein, 627/701, Morumbi, CEP: 05651-901 São Paulo, Brazil; Universidade Federal de São Paulo, Rua Sena Madureira, 1500 - Vila Clementino, 04021-001 São Paulo-SP, Brazil; Santa Casa Misericórdia de São Paulo, Dr. Cesario Motta Junior, 61 - Vila Buarque, 01221-020 São Paulo-SP, Brazil; Instituto de Matemática e Estatística, Universidade de São Paulo, Rua do Matão 1010 - Cidade Universitária, 05508-090 São Paulo-SP, Brazil; LIM44, Universidade de São Paulo, Rua Dr Éneas de Carvalho Aguiar, 255 - Cerqueira César, 05403-000 São Paulo-SP, Brazil

## Abstract

**Introduction:**

Although there is an increase in clinical trials assessing the efficacy of cell therapy in structural and functional regeneration after stroke, there are not enough data in the literature describing the best cell type to be used, the best route, and also the best nanoparticle to analyze these stem cells *in vivo*. This review analyzed published data on superparamagnetic iron oxide nanoparticle (SPION)-labeled stem cells used for ischemic stroke therapy.

**Method:**

We performed a systematic review and meta-analysis of data from experiments testing the efficacy of cellular treatment with SPION versus no treatment to improve behavioral or modified neural scale outcomes in animal models of stroke by the Cochrane Collaboration and indexed in EMBASE, PubMed, and Web of Science since 2000. To test the impact of study quality and design characteristics, we used random-effects meta-regression. In addition, trim and fill were used to assess publication bias.

**Results:**

The search retrieved 258 articles. After application of the inclusion criteria, 24 reports published between January 2000 and October 2014 were selected. These 24 articles were analyzed for nanoparticle characteristics, stem cell types, and efficacy in animal models.

**Conclusion:**

This study highlights the therapeutic role of stem cells in stroke and emphasizes nanotechnology as an important tool for monitoring stem cell migration to the affected neurological locus.

## Introduction

Stroke has ranked recently as the second most common cause of death in the Global Burden of Diseases, Injuries, and Risk Factors Study (2010) [1] and as the third most common cause of disability-adjusted life-years (DALYs) worldwide (2010) [2]. Despite the heterogeneity of global epidemiological data and the measurement bias in places without trained professionals, evidence from developed countries suggests that one out of 20 adults (more than 14 years old) is affected by stroke, and this exceeds the current incidence of acute coronary heart disease. Therefore, stroke constitutes the leading cause of mortality among adults [3].

Conventional clinical management includes percutaneous intravascular interventions and thrombolytic therapy or other medications such as aspirin and behavioral rehabilitation strategies. The wide use of thrombolytic therapy is limited by the narrow time window (within 3 to 4.5 hours after the onset of acute stroke) and serious hemorrhagic complication [4]. Thrombolytic therapy (recombinant tissue plasminogen activator, or rt-PA) is still the most efficient procedure used to restrict neurological damage, although its effectiveness is dependent on and limited to a narrow time window (3 to 4.5 hours after the onset of acute stroke) and the risk of severe hemorrhagic complication. Neuroprotective therapies or other procedures, such as erythropoietin (EPO), N-methyl d-aspartate (NMDA) antagonists, and gamma-aminobutyric acid (GABA), have shown positive results in preclinical stroke trials but no evidence of clinical efficacy [5,6].

Given this therapeutic setting, the use of stem cells or stem cell therapy is emerging as a viable option for neurorestorative stroke, especially in cases where the start time of rt-PA exceeded the ideal. The stem cell therapy promotes neuroprotection and neurorepair because of their ability to produce and secrete neurotrophic factors, and it stimulates the replacement of damaged neurons, enabling a favorable neuroimmunomodulation environment for repair [6,7].

**Figure 1:**
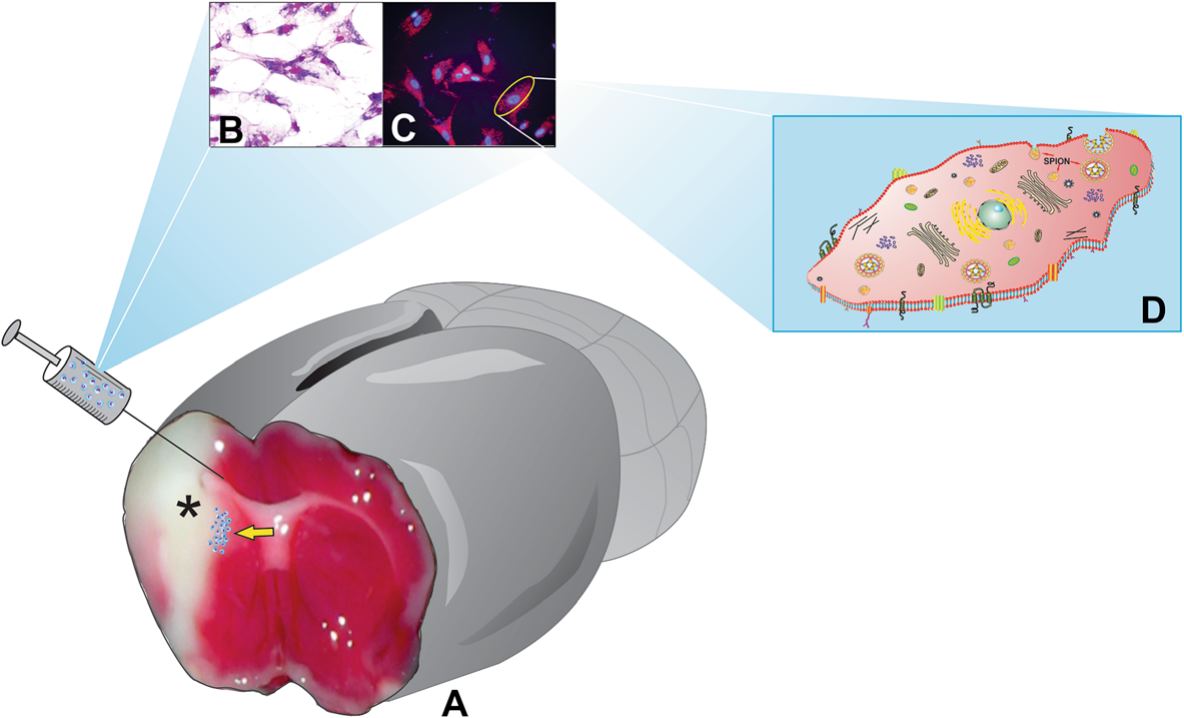
**Mesenchymal stem cell therapy after focal ischemia.** Rat brain schematic figure of grafting process of the mesenchymal stem cells labeled with superparamagnetic iron oxide nanoparticles after focal ischemia. **(A)** Coronary rat brain slice staining with 2,3,5-triphenyltetrazolium chloride after occlusion of the middle cerebral artery. **(B-C)** Umbilical cord of mesenchymal stem cells labeled with superparamagnetic iron oxide nanoparticles, with Prussian blue and rhodamine in fluorescence microscopy, respectively; * focal ischemia. **(D)** Schematic representation of mesenchymal stem cell interactions with nanoparticles.

The route of administration is crucial for the success of stem cell transplantation because tracking and monitoring of grafted cells are necessary, given the minimum concentration of cells required for the surgery as described earlier. Several techniques using nanoparticles—quantum dots, pebbles, and superparamagnetic iron oxide nanoparticles (SPIONs) [8]—have been described to screen cells *in vivo*. The iron oxide and magnetic resonance imaging (MRI) can be used not only to evaluate whether the cells have been successfully engrafted but also to monitor the time course of cell migration in the targeted tissue (Figure 1) [9].

This study evaluated functional outcome in publications of the past decade about stem cells labeled with iron oxide nanoparticles in a preclinical ischemic model. Considering reports included in this review, we sought to provide a comprehensive synopsis of preclinical evidence using various donor cell types, their restorative mechanisms, delivery methods, future prospects, and challenges for translating cell therapies as a functional therapy for stroke in clinical settings.

## Methods

### Search strategy

We included reports between January 2000 and October 2014 that were found in the following databases: Cochrane Library, EMBASE, PubMed, and Web of Science. A Boolean strategy was applied. The following sequence of keywords and Boolean operators (DecS/MeSH) were used: EMBASE: ‘stem cell’/exp OR ‘stem cell’ AND (‘iron oxide’/exp OR ‘iron oxide’ OR nanoparticle) AND (‘stroke’/exp OR stroke OR ‘cerebral ischemia’); PubMed: (((stem cell [MeSH terms]) AND (iron oxide OR SPIO OR nanoparticle)) AND ‘cerebral ischemia’) OR (((stem cell [MeSH terms]) AND (iron oxide OR SPIO OR nanoparticle)) AND stroke); Web of Science: TS = (stem cell) AND TS = (nanoparticle) AND TS = (cerebral ischemia) OR TS = (stem cell) AND TS =(nanoparticle) AND TS = (stroke) OR TS = (stem cell) AND TS = (iron oxide) AND TS = (cerebral ischemia) OR TS = (stem cell) AND TS = (iron oxide) AND TS = (stroke); Cochrane Library: ‘stem cell’, ‘iron oxide’ OR ‘nanoparticle’ AND ‘stroke’.

### Data extraction

Two reviewing authors independently extracted data, screened all references to verify eligibility, and assessed the quality of the trial. Discrepancies in selection of studies and data extraction that appeared between the two reviewers were discussed with a third reviewer and resolved. We undertook a quantitative evaluation of data by using fixed-effect meta-analyses.

### Study selection

**Figure 2:**
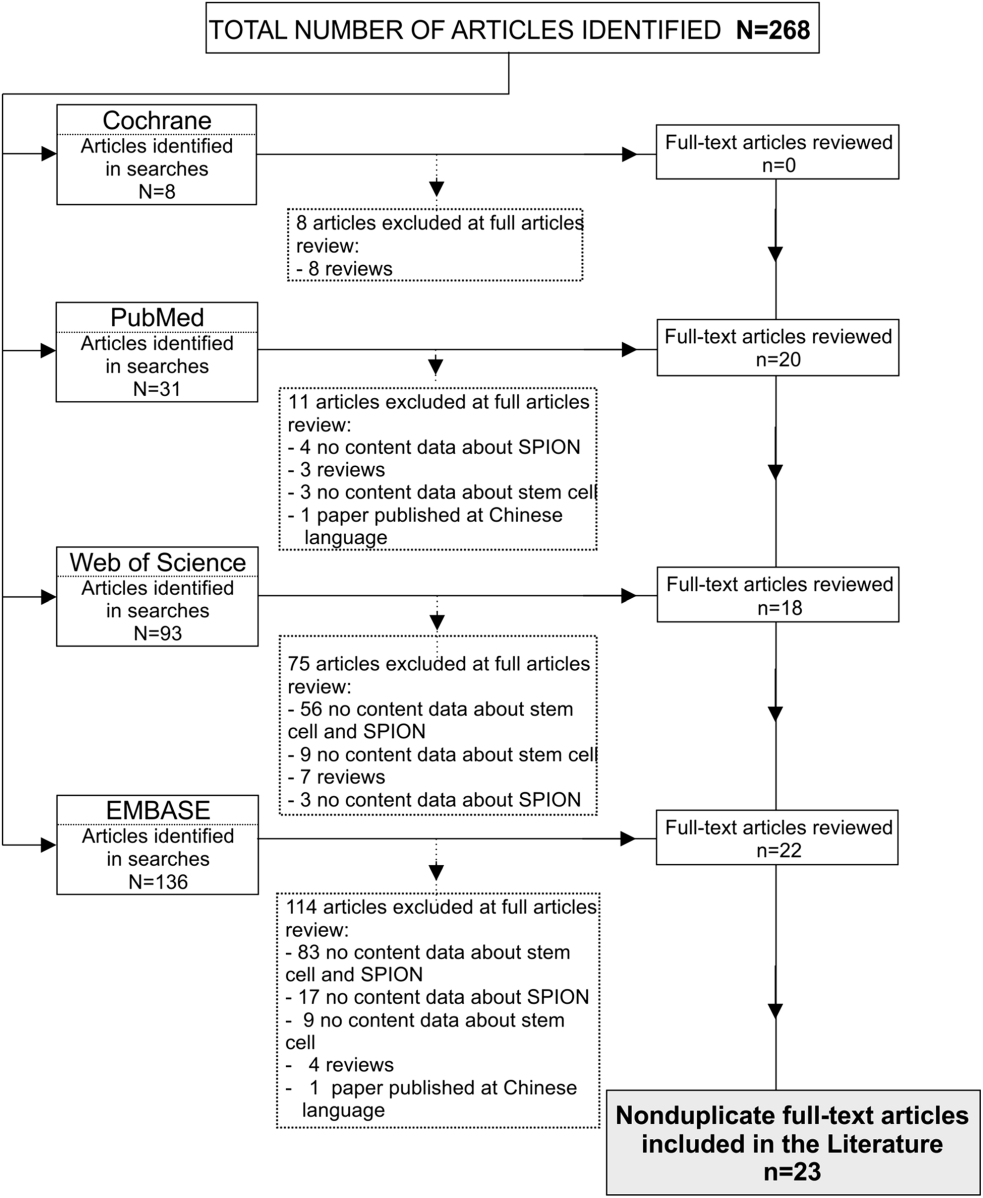
**Flowchart of the article screening process for inclusion in this review.** SPION, superparamagnetic iron oxide nanoparticle.

Studies included were original reports written in English that used stem cells labeled with paramagnetic iron oxide nanoparticles (SC/SPIONs) in stroke preclinical models published between January 2000 and October 2014. We excluded from this review duplicate reports indexed in more than one database, incomplete articles, studies in conferences, congress or symposium format, book chapters, and publications not in English or those not related to nanoparticles of iron oxide or stem cells (Figure 2).

### Statistical analysis

Data were analyzed by applying a meta-analysis approach [10]. Heterogeneity was evaluated by using the I^2^ statistic; a considerable heterogeneity (I^2^ > 50%) was explored by using a fixed-effects model by the free software R version 3.1.0 (Free Software Foundation, Boston, MA, USA).

## Results

In total, 268 articles were identified by the Cochrane Collaboration and indexed in PubMed, Web of Science, and EMBASE. After inclusion criteria were applied, 24 studies were selected (Figure 2). Of these, 20 (83%) were published within the past 5 years (2008 to 2013). Most (52%) of the studies were conducted in Asia, followed by the United States and European countries.

The main characteristics of selected studies are presented in Table 1. In regard to the experimental model adopted, one study [11] used dogs, two studies [12,13] used New Zealand rabbits, three studies [14-16] used mice, and other studies (77%) used rats (200 to 310 g). Experimental methods used were the lacunar method in one study [17] and the photothrombosis method in four studies [18-21] conducted in Belgium. These studies used rose Bengal (10 or 20 mg/kg) for 20 minutes and focused a light beam of 540 nm. In addition, 18 of 19 other studies [11-16,22-33] used the temporary occlusion method of a cerebral vascular bed. Permanent occlusion was reported only in studies by Reddy *et al*. [12], Gutiérrez-Fernández *et al*. [34], Wang *et al*. [35], and Tarulli *et al*. [32]. The remaining studies performed temporary occlusion, which ranged from 5 minutes [13,16,31] to 120 minutes [24,25,28]. The most common vascular beds used were the middle cerebral artery as well as the internal carotid artery of rabbits (New Zealand) [12,34] and the common carotid artery of rats [13].

**Table 1:** **Motor performance after therapy with stem cells labeled by iron oxide nanoparticles in experimental designs of stroke**

**Reference**	**Experimental model**			**SPION**			**Stem cell**				**Behavioral score**	
	**Animal**	**Type**	**Type**	**Type**	**[Fe] μg/mL**	**Source**	**Type**	**Concentration**	**Route**	**Time**	**Sham**	**Lesion**
Wen *et al*. [33] (2014)	Rat (Sprague-Dawley)	MCDA	T (N/A)	In lab	26	Rat	NSC	5 × 10^5^	Str	2 d	N/A	N/A
Shichinohe *et al*. [17] (2013)	Rat (Wistar)	Lacunar	T (N/A)	Resovist	1	Rat	BMSC	5 × 10^5^	Str	7 d	N/A	N/A
Tarulli *et al*. [32] (2013)	Rat (Long Evans)	MCDA	P	MOC07F, Bang Lab.	18,8	Rat (femur and tibia)	BMSC	3 × 10^6^	V	3 d	N/A	N/A
Zhang *et al*. [16] (2013)	Mice (CD-1)	MCDA	T (5 mi)	In lab	5-33	Mice	NSC	5 × 10^5^	IC; V	1 d	N/A	N/A
Liu *et al*. [31] (2013)	Rat (Sprague-Dawley)	MCDA	T (5 mi)	In lab	N/A	Rat	NSC	3 × 10^4^	Str	2 d	N/A	N/A
Lu *et al*. [11] (2013)	Dog (Beagle)	MCDA	T (2 h)	In lab	N/A	Dog (bone marrow)	MSC	3 × 10^6^	IC	2 h	N/A	N/A
Kamiya *et al*. [30] (2013)	Rat (Sprague-Dawley)	MCDA	T (90 mi)	In lab	N/A	Rat (femur and tibia)	MSC	10^7^ (1 mL)	IC	90 mi	N/A	N/A
Riegler *et al*. [13] (2012)	Rabbit (New Zealand)	OAF	T (5 mi)	FluidMag-D, FluidMag-lipid, FluidMag-DEAE, FluidMag, Endorem, Resovist	16-56.0	Rabbit	MSC	10^5^ (300 μL)	V	5 mi	2.9 ± 0.93	1.6 ± 0.38
Detante *et al*. [29] (2012)	Rat (Sprague-Dawley)	MCDA	T (90 mi)	In lab	10.0	Human (bone marrow)	MSC	10^5^ (5 μL)	Str IC	7 d	3.3 ± 1.5	1.4 ± 0.8
Yang *et al*. [28] (2011)	Rat (Sprague-Dawley)	MCDA	T (2 h)	In lab	25	Human (bone marrow)	MSC	6 × 10^5^	IC	14 d	N/A	N/A
Wang *et al*. [15] (2011)	Mice (CD-1)	MCDA	P; T (30 mi)	In lab	N/A	Mice (femur and tibia)	MSC	5 × 10^5^ (1 μL)	Str	0 d	N/A	N/A
Gutiérrez-Fernández *et al*. [34] (2011)	Rat (Sprague-Dawley)	MCDA	P	Endorem	11.2	Rat (femur and tibia)	MSC	2 × 10^6^ (650 μL)	IC V	0 d	3.4 ± 0.89	1.7 ± 0.53
Vandeputte *et al*. [21] (2011)	Rat (Fisher 344)	FOT1	N/A	Resovist	27.9	Rat	rMAPC	10^6^ (1 μL)	Str	1 d	N/A	N/A
Reddy *et al*. [12] (2010)	Rabbit (New Zealand)	ICAO	P	Resovist	11.2	Human (bone marrow)	MSC	10^6^ (1 μL)	IC	4 d	N/A	N/A
		PPA										
Crabbe *et al*. [20] (2010)	Mice	FOT1	N/A	Resovist; Endorem; Sinerem	11.2-27.9	Mice	MSC	10^4^-5 × 10^6^	Str	2 d	N/A	N/A
	Rat (Fisher 344)											
Song *et al*. [27] (2009)	Rat (Sprague-Dawley)	MCDA	T (2 h)	Feridex	11.2	Human	NSC	4 × 10^5^ (1 μL)	Str	1 d	3.6 ± 2.1	2.3 ± 0.42
Daadi *et al*. [26] (2009)	Rat (Sprague-Dawley)	MCDA	T (30 mi; 1 h)	Feridex	11.2	Human	ESC	5 × 10^4^-10^6^ (1 μL)	Str	2 d	11.5 ± 7.2	32.4 ± 12.3
Lee *et al*. [19] (2009)	Rat (Wistar)	FOT2	N/A	MPIO	11.2-27.9	Human	MSC	2 × 10^4^	IC	2 d	N/A	N/A
				Resovist				2 × 10^6^	V			
Walczak et al. [25] (2008)	Rat (Wistar)	MCDA	T (2 h)	Feridex	11.2	Rat (bone marrow)	MSC	10^6^ (2 μL)	V	30 mi	N/A	N/A
Kim *et al*. [24] (2008)	Rat (Sprague-Dawley)	MCDA	T (2 h)	Feridex	11.2	Human	MSC	10^5^	IC	7d	N/A	N/A
Guzman *et al*. [23] (2008)	Rat (Sprague-Dawley)	MCDA	T (1 h)	Feridex	11.2	Mice	NSC	2 × 10^5^ (2 μL)	Str	−7 d	N/A	N/A
Rice *et al*. [14] (2007)	Mice (C57B/6)	MCDA	T (1 h)	Feridex	11.2	Mice	fMSC	5 × 10^2^	IHp	1 d	3.7 ± 0.99	2.3 ± 0.48
								5 × 10^3^	Str			
Jendelová *et al*. [18] (2004)	Rat (Wistar)	FOT2	N/A	Endorem	11.2	Rat	ESC	2 × 10^5^	IC	7 d	N/A	N/A
							MSC	2 × 10^6^	V			
Hoehn *et al*. [22] (2002)	Rat (Wistar)	MCDA	T (1 h)	Sinerem	20.0	N/A	ESC	2 × 3 × 10^4^	Str	14 d	N/A	N/A
									CC			

BMSC, bone marrow-derived mesenchymal stromal cell; CC, corpus callosum; d, day; ESC, embryonic stem cell; fMSC, fat mesenchymal stem cell; FOT1, photothrombosis model (rose Bengal 20 mg/kg, 20 minutes, 540 nm light); FOT2, photothrombosis model (rose Bengal 10 mg/kg, 10 minutes, light 327 to 650 nm; h, hour; IC, intracortical; ICAO, occlusion of the internal carotid artery; IHp, intrahippocampal; MCDA, occlusion of the middle cerebral artery; mi, minute; MSC, mesenchymal stem cell; N/A, not identified; NSC, neural stem cell; OAF, femoral artery occlusion; P, permanent; PPA, ; rMAPC, rat multipotent adult progenitor cell; SPION, Superparamagnetic iron oxide nanoparticles; MCDA, occlusion of the middle cerebral artery; OAF, femoral artery occlusion; ICAO, Occlusion of the internal carotid artery; FOT1, photothrombosis model (rose Bengal 20 mg/ kg, 20min, 540nm light); FOT2, photothrombosis model (rose Bengal 10 mg/ kg, 10min, light 327 - 650nm; T, temporary; P, Permanent; mi, minute; h, hour; N/A, not identified; MOCO7F, MPIO, NSC, neural stem cell; BMSC, bone marrow-derived mesenchymal stroma cell; MSC, mesenchymal stem cells; rMAPC, rat multipotent adult progenitor cell; ESC, embrionary stem cell; Str, intrastriatum; V, endovascular; IC, intracortical; CC, Corpus callosum; IHp, intrahippocampal; fMSC, fat mesenchymal stem cell; d, days.

The labeling cells with SPIONs in most studies [13,14,18,20,24-27,34] were Feridex (or Endorem), four studies [13,19-21] used Resovist, two studies [20,22] used Sinerem, and one study [13] used FluidMag-D, FluidMag-lipid, DEAE-FluidMag, FluidMag-P, and FluidMag-Q. One study [29] used fluorescent iron nanoparticles (excitation 480 nm and emission 250 nm). In addition to the 13 studies that evaluated commercial nanoparticles, four studies [12,15,19,30] evaluated nanoparticles synthesized in the lab: (i) Lee *et al.* [19] used nanoparticles synthesized from co-precipitation and polymerization processes; however, these processes were changed to obtain nanoparticles of different diameters, such as 100 to 750 nm; (ii) Reddy *et al.* [12] used magnetic nanoparticles synthesized by the sonochemical method followed by coating with the Chitosana process; (iii) in the study by Wang *et al.* [15], synthesis occurred in two stages: the first stage generated synthesis of magnetite nanocluster polystyrene (PMNC), and the second promoted a PMNC coat with silica and rhodamine layer.

For cell process 1, the study [21] used adult progenitor cells, two studies [26,27] used neural lineages, and 13 studies used mesenchymal stem cells (MSCs). Of these 13 trials, four did not specify the cell tissue origin [13,18,20,24], four extracted mesenchymal cells from bone marrow [12,25,29,30], one used fetal MSCs [19], and two used stem cells extracted from tibia and femur [15,34].

**Figure 3:**
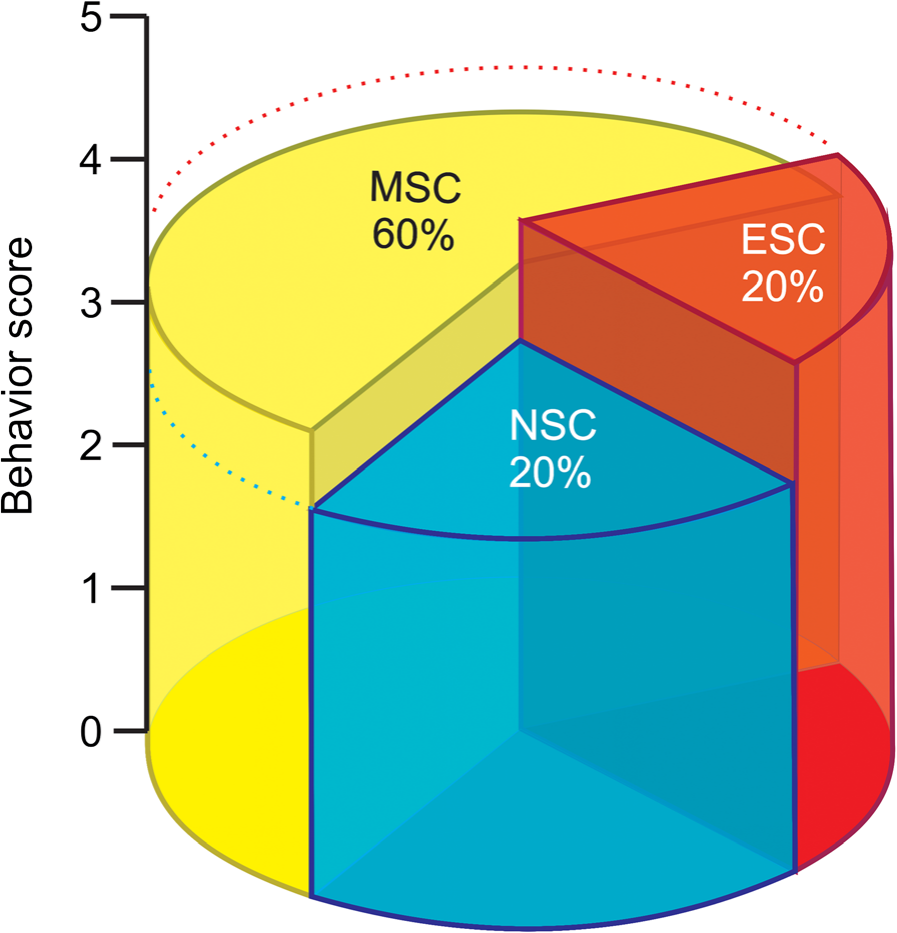
**Functional outcome after stem cell therapy in stroke model.** Three-dimensional pie chart of stem cell distribution with behavior score after rodent focal ischemia. ESC, embryonic stem cell; MSC, mesenchymal stem cell; NSC, neural stem cell.

The majority of cells were from rats [7,18,21,25,30-34]; however, seven studies used human cells [12,19,24,26-29], five studies used mice cells [14-16,20,23], one study used dog cells [11], and one study used rabbit cells [13]. Only one study did not specify cell type [22]. In relation to cell concentration used for labeling with SPION, only four studies reported this information [13,19,25,27]. The study by Walczak *et al*. [25], among the identified studies, used the highest cell concentration (1 × 10^6^), two studies [19,27] used the same cell concentration (5 × 10^5^), and the study by Riegler *et al*. [13] used only 10^4^ cells. These cells were grown mainly in Dulbecco’s modified Eagle’s medium (DMEM) culture [2,14,18,19,22,26,27,34]. Of these seven articles, one [26] used DMEM with F12, another [25] used alpha-minimum essential medium (α-MEM) with F12, and Detante *et al*. [29] used only α-MEM without F12. Only one study [13] used a specific culture for MSCs, and the other four studies [15,20,21,24] did not specify the culture medium used. Of selected studies, only five [13,26,27,29,34] reported behavior score; therefore, three [13,29,34] used MSCs, and two used neural [27] or embryology [26] stem cells (Figure 3).

When it comes to the analysis of the process of cell labeling with nanoparticles or SPIONs, most studies [12,14,20-22,24-26] used transfection agents. Of these studies, six [12,14,20,21,25,26] used poly(l-lysine) (PLL), one [22] used Fugene, one [24] used protamine sulfate as transfector agent, and one [13] used serum deprivation as a process of internalization. The most common SPION concentration in this process was approximately 374 μg/mL, which was identified in five studies [14,18-20,22], but iron concentrations ranged between 0 g/mL [13] and 2,800 g/mL [22] of Fe^2+^. In regard to deployment of the SC/SPION procedure, the selected studies used cerebral application in specific regions such as striatum [14,15,17,20-23,26,27,29,31,33], hippocampus [14], and corpus callosum [22] and in non-specific regions such as intraventricular/cortical regions [11,12,16,18,19,24,28-30,34]. Some studies [13,16,19,25,32,34] conducted cerebral, internal carotid artery implementation in peripheral, femoral artery, and vascular bed [13,18,25,34]. The average time between the ischemic event and therapy with SC/SPION was roughly 3.43 days or 82 hours, but some studies [13,34] underwent implantation immediately after intracortical [34] or endovascular [13]. Another study [22] performed the therapy 14 days after the ischemic event.

**Figure 4:**
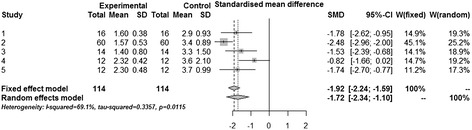
**Forest plot of behavior score of stem cell therapy at preclinical stroke.** CI, confidence interval; SD, standard deviation; SMD, standardized mean difference; W, weight.

The most significant functional recovery was 14 days after implantation of SC/SPION described by Gutiérrez-Fernández *et al*. [34]. Therapeutic efficacy was evaluated during the experimental model (Figure 4) in only five studies [13,26,27,29,34] that conducted behavioral testing based on adaptations of scales of clinical neurology; other studies have evaluated the efficiency of the model by the analysis of MRI and also by histological analysis (Table 2). No significant influence related to route of administration on either structural or functional outcome was seen.

**Table 2:** ***In vivo*** **and** ***Ex vivo*** **image data**

**Reference**		**Magnetic resonance image**					**HistologicaI image**		
	**MF (T)**	**Sequence**	**Weighted images (TR/TE;ms)**	**FOV; MT; ST**	**Results**	**Date (days)**	**Assay**	**Results**	**Date (days)**
Wen *et al.* [33] (2014)	1.5	FSE	T2:2000/100	40; 256×256; 1mm	H+	7,14,21,28,35,42	PB; GFAP MAP2	H+	42
		FFE	T2*:600/18.31						
Shichinohe *et al.* [17] (2013)	7.0	Spin echo	T2:2500/60	30×30; 512×512; 1mm	H+	2,8,14,28,49	HE; TB; GFAP NeuN	H+	N/A
Tarulli *et al.* [32] (2013)	3.0	FSE	T2:8/70	30×30; 512×512; 1mm	H+	7,14	PB	H+	15
Zhang *et al.* [16] (2013)	7.0	N/A	N/A	N/A; N/A;	H+	1,2,3,4,5,6,7	PB	H+	N/A
Liu *et al.* [31] (2013)	3.0	GRE	T2*:2560/6.0	6.0; N/A; 1.6 mm	H+	1, 7,21	PB; BrdU;SOX-2	H+	7,21
Lu *et al.* [11] (2013)	3.0	T2WI	T2*: 5000/60	200;320×320; 2mm	H+	1, 7,14, 21, 28	HE; PB	H+	28
		DWI							
Kamiya *et al.* [30] (2013)	7.0	3D GRE	T2*: 100/10	50×50; 256×256; 5mm	H+	1h, 1, 3, 7	BB; PKH26	H+	7
		T2WI	T2*: 2000/60	50×50; 256×128; 5mm				LL	
Riegler *et al.* [13] (2012)	9.4	3D GRE	T2:6000/105	70×70; 512×512; 1mm	H+	24h	PB	H+	21
			T2*:6000/105						
Detante *et al.* [29] (2012)	2.35	SE-DW	DW:2000/80	N/A; 234×234; 1mm	H+	1, 15, 28	GFAP	H+	1, 15, 28
			T2*:400/25					Tr+	
Yang *et al.* [28] (2011)	3.0	MPGR	T2*:596/16	292×290; 0.7mm	H+	1,15	GFAP;PB	H+	N/A
Wang *et al.* [15] (2011)	3.0	N/A	T2: 5840/104	45×45; 256×256; 1.5mm	H+	1, 3	GFAP;PB	H+	1, 7, 30
					Tr+			Tr+	
Gutiérrez-Fernandez *et al.* [34] (2011)	7.0	RARE	T2	N/A	H+	24h, 14	NeuN	H+	N/A
					LL		GFAP	LL	
							VEGF		
Vandeputte *et al.* [21] (2010)	9.4	N/A	T2: N/A	N/A	H+	24H, 2-18	MAP2	H+	N/A
								Tr+	
Reddy *et al.* [12] (2010)		Turbo Spin echo	T2: 2128/80	230×230; 700×625, 1mm,	H+	4, 16	PB	H+	16
	3.0		T2: 2548/80	80×80;94×94; 1.5mm				Tr+	
			DW: 4763/50	80×80;94×94; 1.5mm					
Crabbe *et al.* [20] (2010)	9.4	2D MSME	T2: 6000/10; DW: 1500/27	4.0×4.0; 156×156; 0.8 mm	H+	12h, 10	MAP2	H+	N/A
				N/A; N/A; 1mm				Tr+	
Song *et al.* [27] (2009)		3D spin echo	T2: 3500/80	60×60; 256×160 2.0mm	N/A	N/A	N/A	N/A	N/A
							PB		
	1.5		T2:50/20	80× 80; 256×160; 2mm	H+	−1, 1, 3, 7,14, 21, 28	NeuN	H+	1, 28
							GFAP	Tr+	
							BrdU		
Daadi *et al.* [26] (2009)	7.0	2D – spin echo	T2: 4000/82,5	5cm; 256×256; 0.6 mm	H+	2, 7, 14, 28, 60	hNSC	H+	60
					LL		GFP	LL	
							NeuN		
Lee *et al.* [19] (2009)	1.5	Turbo spin echo	T2: 2000/81	5cm; 512×512; 1.5mm	H+	−1, 1, 5, 12	hVim	H+	N/A
		GRE	T2*: 280/20				fMSC	Tr+	
Walczak *et al.* [25] (2008)	4.7 or 9.4	3D Spin echo	T2: 1300/98	34×22×11; 128×64×3; 0.35mm	H+	−1h, 2h-1	BrdU	H+	N/A
		GRE	T2*: 300/5	10×16; 128×128				Tr+	
Kim *et al.* [24] (2008)	4.7	3D Spin echo;	T1; N/A	4×3	H+	2, 7-70	hMSC	H+	Cell in core of lesion
		RARE; Flash	T2: 600/14	256×192; 1mm				Tr+	
			T2*:758/30						
Guzman *et al.* [23] (2008)	4.7	Spin echo	T2:2500/45	40; 256×256; 1mm	H+	−4, 3, 7,24	AP BrdU; GFAP; βTubulin	H+	N/A
		3D GRE	T2*:600/5					Tr+	
Rice *et al.* [14] (2007)	7.0 or 9.4	Spin-echo multislice	T2: 1,0; N/A	3.5cm; 128×128; 1mm	H+	24h, 14	fMSC	H+	N/A
							GFP	Tr+	
Jendelove *et al.* [18] (2004)	4.7	Turbo spin echo	T2:2000/42.5	3,5cm; 256×256; 0,5mm	H+	14-49	MSC*	H+	N/A
							ESC	Tr+	
							GFP		
Hoehn *et al.* [22] (2002)	7.0	2D Multislice	T2: 200/20	20×12×10 256×256×128; 0.5-0.7mm	H+	6, 8, 11, 16	ESC	H+	N/A
		3D Flash					GFP	Tr+	

2D, two-dimensional; 3D, three-dimensional; AP, acidic protein; BrdU, bromodeoxyuridine; DWI, diffusion weighted imaging; ESC, (mouse) embryonic stem cell; FFE, fast field echo; fMSC, fetal mesenchymal stem cell; FOV, field of vision; FSE, fast spin echo; GE, gradient echo ; GFAP, glial fibrillary acidic protein; GFP, green fluorescence protein; GRE, gradient echo; h, hour; HE, hematoxylin and eosin; hMSC, human mesenchymal stem cell; hNSC,human embryonic stem cell-derived human neural stem cell; H+, homing (migration to target site); hVim, human vimentin antibody; LL, loss lesion; MAP2, microtubule-associated with protein 2; MF, magnetic field; MPGR, multiplanar gradient recalled acquisition in the steady state; MSC, rat bone marrow stromal cell; MSME, 2D-Multislice-multiecho ; MT, matrix; N/A, not identified; NeuN, neuronal nuclei; PB, Prussian blue; SE, diffusion-weighted; ST, thickness; RARE, rapid acquisition with relaxation enhancement; T, Tesla; T2WI, -weighted magnetic resonance imaging; T2*, star weighted imaging; TB, Trypan blue; TE, echo time; TR, repetition time; Tr+, tracking (possibility of cellular trace).

For the functional outcome, because the Cochran Q test has a low power when the number of studies is small, we consider the I^2^ statistic to evaluate the heterogeneity of studies. Considering the raw mean difference, we obtained I^2^ = 19.6% (confidence interval (CI) = 0% to 83.3%, *P* = 0,2898), indicating that the studies were homogeneous. Therapy was considered effective, as the combined average difference observed was −1.6255 (CI = −1.8923 to −1.3588, Z statistic = −11.9446, *P* <0.0001). However, because of experimental methodological differences, we also analyzed the standardized mean differences considering the pooled standard deviation of two groups (Figure 4). In this analysis, the heterogeneity between studies was evident: I^2^ = 69.1% (CI = 20.7% to 88%, *P* = 0.0115). However, the conclusions had the same raw mean difference because the combined standardized mean difference observed in fixed effect model was −1.9161 (CI = −2.2383 to −1.5939, Z statistic = −11.6564, *P* <0.0001). Despite the high heterogeneity among studies on the effectiveness of cell therapy in the cerebrovascular accident model, the analysis indicated a significant neuroprotective effect.

All selected studies [11-34] evaluated the cell homing by using MRI and histological analyses to validate anatomical and functional improvement from results in image evaluation (Table 2). Overall, selected studies regarding MRI found SC/SPIONs homing to ischemic area in several time points and routes. In MRI, the magnetic field ranged from 1.5 T [19,27,33] to 9.4 T [13,14,20,21,25]. The studies used several protocols of sequence and weighted and thickness images, and the most widely used was a T_2_-weighted three-dimensional spin echo image of 1 mm.

To assess the extent of injury caused by the induced ischemic stroke, nine studies [11,16,17,19-21,27,30,34] were conducted by using the hematoxylin-and-eosin procedure. Results of microscopic analysis of the lesion extent were consistent with findings obtained in the analysis by MRI. Location and extent of injury did not differ considering the experimental model. However, the evaluation of agreement between histology and imaging examination indicated that only three studies [26,30,34] found a decrease in lesion area treated with stem cells. Kamiya *et al*. [30] and Gutiérrez-Fernández *et al*. [34] agreed in the short interval between the induction of ischemic stroke and transplantation; that is, they performed the transplantation 90 minutes after induced ischemic stroke; however, Daadi *et al*. [26] carried out the transplantation 2 days after induced ischemic stroke. In the study by Gutiérrez-Fernández *et al*. [34], lesion area reduction was independent of the route of administration observed, as Daadi *et al*. [26] and Kamiya *et al*. [30] administrated stem cells only in the lesion area.

The homing process of cells used as a therapy was measured in 13 studies [11,12,14,16,18,19,21,24-27,32,33] by Prussian blue staining. Correlation analysis between the location of labeled cells on MRI and location shown on histological analysis indicated agreement between the analyses as well as the homing of positive cells to the area of interest.

In addition the positive cells homing reached the injured organ viability by Prussian blue staining, three studies [14,18,26] assessed expressions by using the green fluorescent protein (GFP) and in all cases the sites indicated by MRI were in agreement. These results confirmed the location of cells and the maintenance of cell viability. Changes in the microenvironment of the lesion reported by four authors [25,27,31,34] that observed increase labeled for bromodeoxyuridine (BrdU) and cell proliferation process. The study by Walczak *et al*. [25] reported an increase that was positive for BrdU in the perivascular region at the first day after transplantation, and for 10 days after transplantation, positivity was maintained, including more distant regions of the vascular bed. However, despite remaining positive for BrdU, the signal intensity of labeled cells was not maintained in resonance examination. Besides the BrdU labeling, two authors [27,34] conducted labeled for the expression of glial fibrillary acidic protein (GFAP), which is a marker of increased activity of glial cells, especially astrocytes and neuronal nuclei marker (NeuN). In addition, these two studies [27,34] reported increased expression of markers used after transplantation of MSCs, thus indicating increased cell proliferation, glial activity, and preservation of the injured area. In the study by Gutiérrez-Fernández *et al*. [34], animals treated with MSC markers regardless of the route of administration of labeled cells expressed an increase in addition to BrdU compared with control groups (137 ± 9.9 versus 51 ± 9.2; *P* <0.05). There was also an expression of increase in NeuN, GFAP, and vascular endothelial growth factor (VEGF) after 14 days of transplantation in the penumbra area that contributed toward reducing the injured area and inflammatory markers such as tumor necrosis factor-alpha (TNF-α) and interleukin-6 (IL-6).

These results appear to correlate with an improvement in neurological scores observed in transplanted animals (3.4 ± 0.89 versus 1.7 ± 0.53; *P* <0.05). The correlation between increased tissue protective factors in transplanted animals and improved neurological scores was also found in the study by Detante *et al.* [29] that reported an increase of GFAP in animals that received MSCs and also an improvement in neurological scores in comparison with the control group (3.3 ± 1.5 versus 1.4 ± 0.8; *P* <0.05), thus confirming the results of Gutiérrez-Fernández *et al*. [34]. Although some studies [15,17,33] did not conduct behavioral assessment, others found an increase in the expression of GFAP and increased microtubule-associated with protein 2 (MAP2), which indicate an improvement in penumbra area.

## Discussion

All selected studies with behavioral tests reported functional improvement associated with the presence of the MS/SPION complex in ischemic area and neurorepair histological changes. The meta-analysis of these studies showed that MS/SPIONs were efficient for treatments, although few studies applied neurological score or behavior tests. Recent reviews [35-37] also observed therapeutic efficacy of stem cells in several preclinical models of stroke but recognized that many fundamental questions related to cell characterization, cell dosage, cell fate, biodistribution, safety indices, outcome measures, and so on are critical for the successful development of a cell product. The labeling process of stem cells with iron oxide could increase cytotoxicity, but improvement of in vivo homing and tracking image techniques of cells.

### Could stem cells influence neurorepair after stroke?

Stem cells come from various sources, and although they share some common properties, they also differ in many respects and behave differently in terms of their rate of differentiation, trophic factor secretion, and their stimulation of endogenous processes when in a pathologic environment [38]. The key source and type of stem cells of the selected studies were human (bone marrow) and mesenchymal. Although the study by Daadi *et al*. [26] had a better behavior status, using neural embryonic stem cells, the majority of studies used MSCs (60%), some studies used neural stem cells (20%) or embryonic stem cells (20%), and no studies compared the different cell types in the same experiment (Figure 3).

The dose or concentration, route, and fate cells of samples of ranged from 5 × 10^2^ (Rice *et al*. [14] 2007) to 10^7^ (Kamiya *et al*. [30] 2013), and intrastriatal was the main route used. A recent meta-analysis [37] on preclinical studies of mesenchymal stromal cells for ischemic stroke observed that the range of MSCs was from 3.5 × 10^4^ to 4.3 × 10^7^, and intravenous was the main route reported in studies. Yavagal *et al*. [39] evaluated the dose and route of MSCs after 1 hour or 24 hours of ischemic injury per 1 hour of occlusion of the middle cerebral artery, and observed that intra-arterial administration of 1 × 10^5^ MSCs after 24 hours of stroke was more efficient in ameliorating neurological deficits in rodent cerebral ischemia, narrowing change of blood flow, reducing infarct volume, and improving functional status. All selected studies found positive cell tracking of SC/SPIONs by magnetic resonance and histological methods for penumbra area after several routes and times of grafting. Other studies [40,41] and systematic reviews [35,37] also observed several studies with positive stem cell homing to penumbra area after 7 days of stroke. The grafting route can influence the time point and the biodistribution of stem cells and further the aggregation process [37] as previously discussed.

### Stem cell biology of repair in preclinical ischemia model

Among all the lines of stem cells used therapeutically, MSCs can express neuronal markers *in vivo* as well as trophic factors such as brain-derived neurotrophic factor (BDNF), glial-derived neurotrophic factor (GDNF), VEGF, neurotrophin-3 (NT3), and fibroblast growth factor (FGF), and thrombospondins are secreted by MSCs in response to the local microenvironment. These factors, along with their stimulation of neurogenesis and angiogenesis immunomodulation, promote functional recovery [42]. In this sense, these stem cells also stimulate astrocytes, that this past years has been used as a therapeutic target, because its role in maintaining neuronal function and effective endogenous repair [43]. Most of the selected studies [11,18,19,21,24-27,32-34] observed an increase of trophic factors, reduction of the area of injury, inflammatory markers such as TNF-α and IL-6, increase in protein expression, proliferative activity, and increased activity of glial cells.

### Iron toxicity in stem cells

Although reports evaluated the adoption of methods to assess the ability for differentiation and presence of cytotoxicity, the description of these methods and their results are brief and sometimes treated as irrelevant. This agrees with an understanding that such assessments are not the central idea of the study, because this is a strategy to validate the quality of the cells after labeling with SPIONs, which validate the use for the evaluation of *in vivo* cell tracking and homing.

The absence of reported cytotoxicity may result from the adoption of methods already established for cultivation and labeling of the cells. A review by Arora *et al*. [44] reported a study that aimed at standardizing cell labeling with SPIONs, and indicated a low incidence of labeled cells in cytotoxicity because of the concentration of SPIONs, number of cells to be standardized, SPION relationship/cell type, method of internalization, and marking time. This confirms the findings observed in the selected articles. The low incidence of cell death in *in vitro* cellular genotoxicity and lack of changes in differentiation ability, when contemplating the need for biocompatibility of nanomaterials, contribute to their promising applicability as a contrast agent in stem cell studies [45]. As the selected studies focused on assessing the ability of mapping *in vivo* homing, cell tracking, and therapeutic potential, the approach of inducing toxicity remained in the background, and was guided by methodological descriptions, which do not allow full clarification of the interaction effects of SPIONs with cellular structures, until the standardization and safety described are achieved. Therefore, because of the brief description of investigation of the cytotoxic effects on marking with SPIONs found in selected studies, our commitment was to further discuss this subject.

Our study has limitations as the internal validity of choice in select only data published in three major database and Cochrane library. In addition, we used an observational approach rather than an experimental one, which enables one to report only associations rather than causation. Although our search strategy was designed to be exhaustive, it is possible that some published studies were missed; nonetheless, our study is likely to have captured the majority of reports in this field and represents the most complete review to date of the use of SC/SPIONs in experimental stroke.

Safety represents a critical concern before stem cells are allowed to be extensively used in clinical settings. Recently, a meta-analysis [38] of clinical trials searched in MEDLINE and EMBASE and by the Cochrane Central Register of Controlled Trials (June 2014) did not detect associations between MSC treatment and development of acute infusional toxicity, organ system complications, infection, death, or malignancy. Notwithstanding these important caveats, our analyses provide a support for some hypotheses regarding the biology of stem cell-based therapies. There is paucity evidence of use of coils with SPION to cell tracking, but this evidence show up the best ‘future’ outlook in neurological clinical stem cell therapy, because it reduces iron concentration and assists the cell tracking to the brain damage area, and modulates electrophysiology of this area to repair. This review did not identify the use of coils in the selected articles, but explores the evidence regarding the therapeutic use of SPION in preclinical models of stroke.

## Conclusions

Selected studies show great promise for cell transplantation as a new therapeutic modality for stroke. Beneficial effects of stem cells might include neuroprotection, angiogenesis, inflammatory, and immune responses. Although animal studies and reviews demonstrated that impaired neural function has been significantly improved after administration of various stem cells, few clinical trials have found similar benefits. A better understanding of the mechanisms of stem cells for treatment of stroke will help resolve the heterogeneity of results. In the future, stem cells combined with gene therapy or rt-PA will play an important role in experimental and clinical settings.

## Abbreviations

BrdU: bromodeoxyuridine
CI: confidence interval
GFAP: glial fibrillary acidic protein
IL-6: interleukin-6
MeSH: Medical Subject Headings
MRI: magnetic resonance imaging
MSC: mesenchymal stem cell
NeuN: neuronal nuclei (marker)
rt-PA: recombinant tissue plasminogen activator
SC/SPIONs: stem cells labeled with paramagnetic iron oxide nanoparticles
SPION: superparamagnetic iron oxide nanoparticle
TNF-α: tumor necrosis factor-alpha
VEGF: vascular endothelial growth factor
